# Design of a Mobile Low-Cost Sensor Network Using Urban Buses for Real-Time Ubiquitous Noise Monitoring

**DOI:** 10.3390/s17010057

**Published:** 2016-12-29

**Authors:** Rosa Ma Alsina-Pagès, Unai Hernandez-Jayo, Francesc Alías, Ignacio Angulo

**Affiliations:** 1 GTM—Grup de recerca en Tecnologies Mèdia, La Salle—Universitat Ramon Llull, Quatre Camins, 30, Barcelona 08022, Spain; falias@salleurl.edu; 2 DeustoTech—Fundación Deusto, Avda. Universidades, 24, Bilbao 48007, Spain; unai.hernandez@deusto.es (U.H.-J.); ignacio.angulo@deusto.es (I.A.); 3 Facultad Ingeniería, Universidad de Deusto, Avda. Universidades, 24, Bilbao 48007, Spain

**Keywords:** END, noise mapping, hardware platform, connectivity, acoustic sensing, signal processing, dynamic measurement, ubiquitous, smart city

## Abstract

One of the main priorities of smart cities is improving the quality of life of their inhabitants. Traffic noise is one of the pollutant sources that causes a negative impact on the quality of life of citizens, which is gaining attention among authorities. The European Commission has promoted the Environmental Noise Directive 2002/49/EC (END) to inform citizens and to prevent the harmful effects of noise exposure. The measure of acoustic levels using noise maps is a strategic issue in the END action plan. Noise maps are typically calculated by computing the average noise during one year and updated every five years. Hence, the implementation of dynamic noise mapping systems could lead to short-term plan actions, besides helping to better understand the evolution of noise levels along time. Recently, some projects have started the monitoring of noise levels in urban areas by means of acoustic sensor networks settled in strategic locations across the city, while others have taken advantage of collaborative citizen sensing mobile applications. In this paper, we describe the design of an acoustic low-cost sensor network installed on public buses to measure the traffic noise in the city in real time. Moreover, the challenges that a ubiquitous bus acoustic measurement system entails are enumerated and discussed. Specifically, the analysis takes into account the feature extraction of the audio signal, the identification and separation of the road traffic noise from urban traffic noise, the hardware platform to measure and process the acoustic signal, the connectivity between the several nodes of the acoustic sensor network to store the data and, finally, the noise maps’ generation process. The implementation and evaluation of the proposal in a real-life scenario is left for future work.

## 1. Introduction

Nowadays, more people live in urban than in rural areas, representing in 2010 50.5% of the world’s population for the first time in history [1]. It is expected that this tendency will continue since all of the world’s population growth will take place in urban areas in the next four decades according to the United Nations [1,2]. This reality poses new challenges to authorities so as to guarantee the efficient use of the resources of those urban areas and the quality of life of their inhabitants through better services management, which requires significant changes in governance, decision-making and developing specific action plans [3]. To this aim, a technological revolution is driving the change of most of the urban cities under the umbrella of the so-called smart city or smart region paradigms [4,5]. However, the transformation of any city into a smart city is a long and complex process [3], which should take most of the experiences and best practices of initiatives already developed previously.

In this sense, city managers should take note of the results obtained by a recent large-scale study in Europe, which has revealed significant adverse impact of environmental noise on the health and longevity of the inhabitants [6]. Since noise mapping has been evaluated as an answer to this problem [7], the acoustic pollution issue has been included within the smart city paradigm, due to its negative impact on the quality of life of citizens.

The smart city revolution, together with the definition of the Environmental Noise Directive 2002/49/EC (END) [8] and the consequent strategic noise mapping assessment (CNOSSOS-EU) [9], both from the European Commission, has encouraged national, regional and local authorities to address the negative impact of citizens’ quality of life in terms of noise pollution. Several studies can be found in the literature about the implementation of the END directive and its impact on health. In a recent work [10], the road traffic noise exposure for 2012 is detailed based on the END. Other works focus on the END proposal for noise mapping, studying whether it is accurate enough for people exposure estimation [11] or for epidemiological studies [12]. For instance, the evaluation of the analyzed information can lead national road authorities to optimize the installation of noise-reducing pavements or noise barriers where required, as well as evaluating the noise exposure and reduction when these plans are implemented [13]. Despite several studies have already implemented the END, the first years of its application have been characterized by the lack of a common method to perform the noise mapping, which have made results’ comparison almost unfeasible. This problem has been recently solved by the publication of the annex to the Commission Directive 2015/996 [14], which settles common methods that the Member States will have to use from 31 December 2018.

The European Commission (EC) has promoted the research in those topics, by funding working groups like FONOMOC [15] in the framework of the European cities association EUROCITIES [16], constituted to share experiences and knowledge across European cities. From these work groups, several conclusions and future work road-maps have been generated, e.g., as reported in [17], where the mobility in urban areas is studied from different perspectives and several suggestions of improvement are detailed through diverse technical measures and smart or innovative solutions.

In terms of European funding schemes focused on environmental issues, several LIFEprojects have been funded during the last few years to address the noise pollution consequences: from its diagnosis, through the improvement of citizens’ involvement, to the development of specific solutions. The recently ended Harmonica project (LIFE10 ENV/FR/000211) [18] has developed new tools to give the public better information about environmental noise and to help local authorities make the right decisions in fighting noise pollution [19]. On the other hand, the DYNAMAPproject (LIFE13 ENV/IT/001254) [20] aims to develop a dynamic noise mapping system able to detect and represent in real time the acoustic impact of road infrastructure based on a static network of acoustic sensors, reducing the cost of the periodical actualization of the noise maps [21,22]. The latter builds on the conclusions obtained from the SENSEable PISA project [23,24], which proposed collecting the information describing not only the urban environment, but also some aspects of the social behavior of the citizens, to study possible relationships with public health, mobility or pollution. Regardless of the the dramatic improvement that these kinds of systems entail in terms of having a dynamic picture of the noise pollution in the city, their main drawback is that they are static, i.e., they are designed and adjusted once and deployed in specific locations from that moment on. Hence, if there is some significant change in the urban area environment (e.g., super-blocks in Barcelona [25,26]), the deployed system should be redesigned and adapted appropriately to the new operational context.

Nowadays, the European Union (EU) authorities are proposing cities to make a step beyond current approaches and use noise mapping to improve the strategy of urban sound planning [27]; this indication is slowly being acquired by local authorities and being integrated with their environmental noise policies. Noise mapping is used together with soundscape analysis and sound level measurements to follow the management guidelines of areas with good environmental noise quality [28]. To this aim, the SONORUS project [29] is focused on the training of professionals for the integration of urban sound planning in the urban development process of cities, with the final goal of reducing the noise levels.

In order to face the main drawback of static networks, we propose the development of a low-cost ubiquitous sensor network for real-time noise monitoring using urban bus routes. This proposal builds on the idea that urban traffic noise levels can be measured on certain city routes by means of the implementation of a mobile sensor network installed on public buses. From the gathered data, a noise map is dynamically calculated and published right after to inform citizens and authorities. After describing the design of the proposal, we discuss the main challenges it faces, as the mandatory noise cancellation of the sensing vehicle to avoid biasing the noise map computation, the development of low-cost ubiquitous hardware platform and its connectivity to provide the measured data to the cloud, the bus trajectory to collect the most suitable data and, finally, the data gathering in the cloud to tailor an integrated noise map. Finally, it is worth noting that the implementation and evaluation of the proposal in a real-life scenario are not addressed in this work, since these are left for future studies.

This paper is structured as follows. In Section 2, the state of the art of dynamic acoustic urban sensing applications, considering both static acoustic networks and participative sensing approaches, is presented. In Section 3, we compare several low-cost hardware platforms and their connectivity. Section 4 discusses the challenges of the proposal in terms of signal processing, hardware platform and network design. Finally, Section 5 resumes the key characteristics that should be taken into account for the design of a bus-based mobile acoustic urban sensor network to map the urban noise dynamically.

## 2. State of the Art of Dynamic Acoustic Urban Sensing

Traditional noise measurements in cities have been mainly carried out by professionals that record and analyze the data in a certain location typically using certified sound level meters [7]. This approach is hardly scalable when it comes to tackling the current demand for more frequent noise level measurements in both time and space (thus, more measures and in more places). In the last decade, several approaches for monitoring environmental noise have been proposed [30]. Their main goal has been developing less expensive and smaller hardware solutions, but assuring the reliability of the acoustic measure, as well as allowing the scalability of the system through the improvement of network data communications.

In this section, we describe representative approaches developed to automatically measure the noise levels of the cities in order to tailor noise maps [30]. The classification takes into account how the measure was performed, assuming that all of them have been designed to obtain a dynamic noise measure, where the term ‘dynamic’ refers in all this paper to a system that updates its measurement frequently in time. Static acoustic urban networks have been developed in some cities to provide automatic monitoring based on fixed sensor networks placed in specific locations. Finally, some mobile platforms designed to perform acoustic sensing are also described. On the one hand, some of them have conducted the measurements on vehicles, taking advantage of their ubiquity. On the other hand, some projects have asked citizen to become contributors to the city sensing system thanks to the democratization of technology; in particular, smartphones.

### 2.1. Static Acoustic Urban Sensing

In this section, several static noise sensing platforms and their applications are summarized, focusing exclusively on those whose main focus is the monitoring of environmental noise on specific locations in urban areas.

In [31], a project designed for the monitoring of the traffic noise in Xiamen City (China) is presented. The designed system considers noise meters, ZigBee technology and GPRS communication. In [32], the authors prefer to use a customized noise level meter, which is designed to remove the burden of computational and energy-expensive operations from the sensor node and process them in the cloud. In [33], the RUMEUR (Urban Network of Measurement of the sound Environment of Regional Use) network developed by Buitparif is detailed. The sensor network includes both high accuracy equipment for critical places, like airports, where the focus is to obtain detailed acoustic information due to the intense noise environment, together with less precise measuring equipment in other locations where the goal is only the updating of the noise map. In [34], the deployment of an acoustic sensor network based on the FIWARE platform is described. The information collected is used to create quasi-real-time dynamic noise and event maps, as well as for identifying the sound sources. The applications of the project are focused on surveillance, and they range from localizing fire to finding people in distress.

Another related field of study is Wireless Acoustic Sensor Networks (WASN) [35], enabled by the advances in the technology and the availability of small, low-cost and smart sensors that result in deployable nodes of WASN. They are usually applied to sensing remote areas where power connections are difficult or even impossible to reach. Despite most of the applications in this field being found in underwater [36] or environmental situations [37], several smart city applications can be also found in the literature. For instance, in [38], an ad hoc wireless sensor network system that detects and accurately locates shooters in the city is detailed. Thus, in this case, not only the specific type of sound is identified, but also the precise place of the shooter. In Section 2.3, another wireless sensor network is presented [39], but it is used as a mobile sensor network for participative sensing. For more references related to this field and, in particular, about signal processing-related issues, the reader is referred to [40] and the references therein.

The last group of static acoustic urban sensing systems is based on custom-built sensor networks usually designed to be very economical and autonomous, with low power consumption, so they can be installed pervasively. Two of the projects using this kind of network are the IDEA (Intelligent Distributed Environmental Assessment) project [41] and the MESSAGE (Mobile Environmental Sensing System Across Grid Environments) project [42]. They are based on a single-board computer with low computational capacity, working with a low-cost sound card and microphones. This hardware choice allows the deployment of large sensor networks due to the economic cost of each node, besides allowing the collection of relevant environmental data from several critical locations in the city. In the IDEA project [43], a cloud-based platform is developed integrating an environmental sensor network with an informative web platform and pretends to measure noise and air quality pollution levels in urban areas in Belgium. This is also the base idea of the DYNAMAP (DYNamic Acoustic MAPping) project [21,44], aimed at the deployment of two low-cost acoustic sensor networks, one in Rome and the other in Milan, as a step ahead from the preliminary results obtained by the SENSEable project deployed in the city of Pisa (all of them in Italy) [24].

### 2.2. Mobile Acoustic Urban Sensing

Some acoustic urban sensing approaches have built on mobile urban sensing systems during the last years. The mobile concept refers here to the execution of the measurement; thus, in this section we are focusing on those platforms that allow changing the place of measurement. The goal of these systems in comparison with the previous static urban sensing approaches is that the potential area to be sensed is widened by means of the sensor network ubiquity. One of the challenges that these systems face is the spatial resolution of urban noise maps when the measures are collected through mobile devices. In [45], the authors detail the relevance of different interpolation techniques in mobile sensing in comparison with the usual spatial interpolation techniques (such as inverse distance weighting [46] or kriging [47]).

One of the first experiences for this goal is detailed in [48], where a Mobile Sensing Unit (MSU) associated with a GPS position is used, allowing the ubiquitous sensing of larger areas. The Seoul ubiquitous sensing project conducted a wide range of tests across several city locations with a reduced set of sensors. The MSUs were even installed in cars and buses moving around the city following repetitive circuits. These nodes measured temperature and humidity and also noise level; but no signal processing is detailed in the description of the system oriented toward the cancellation of any noise, but traffic noise; especially the one produced by the MSU car or bus.

In [49], an array of sensors mounted on a vehicle driving along the streets of the city is proposed in order to acquire measurements from different locations quickly. The goal of that piece of research is estimating the locations and the power of the stationary noise sources of the places of interest. For this purpose, the data gathered by the array is post-processed before plotting the several sources in the noise map.

The work in [50] focuses on the study of low density roads. The introduced approach includes mobile [51] and fixed noise monitoring platforms, obtaining data about traffic, noise and air pollution. The proposal is based on performing the mobile measurements by bicycle, which provides a new view of the local variability of noise and air pollution based on computing the differences of measurements along road segments [52]. This proposal is easily applicable to other cities to monitor noise and air pollution. One of the strong assets of this proposal is that it is prepared to measure more parameters, but audio; so, a wide amount of environmental information can be collected. In [51], also black carbon concentration measurements are performed, so potential health risks for citizens can also be estimated. Not only estimating environmental parameters, but mobile acoustic sensors can also be used for other applications, but noise monitoring, e.g., for soundscape identification [53], where they derived geo-information from audio data, to determine the position of a mobile device in motion by means of the audio data recorded by its built-in microphone.

### 2.3. Participative Urban Sensing

Finally, the last urban sensing method used nowadays is based on participative measurements; that is, considering the so-called citizens as sensors paradigm [54]. In this case, tools and protocols have to be predefined to allow information integration of all of the individual measurements (e.g., when uploading geolocalized photos in Google Maps, reporting traffic accidents in the Waze application, etc.).

When it comes to measuring city noise levels, two main participative sensing approaches can be found in the literature, depending on the tools used by citizens: (i) those considering citizens’ tools, usually through mobile applications installed in their smartphones, as described in [55]; and (ii) those where citizens are provided with specific tools to be used for conducting the measurements, as in the project CITI-SENSE [56]. These tools and observation protocols are defined to allow citizens to collect simultaneous objective and subjective data of the environmental sound by themselves; each project develops products and services to perform the tests. For example, in the CITI-SENSE project [57], it includes a mobile application, an external microphone with a windscreen to protect the microphone of the smartphone and other external equipment, connected via Bluetooth to the smartphone, to measure climatic conditions.

In [39], the authors reviewed the approaches already presented by the wireless sensing research community to assess noise pollution using both acoustic sensor nodes and mobile phones. Since then, several works have been published dealing with the integration of the results of the individual measurements. This approach increases the number of potential measurements to be evaluated in the integration of the data, which is usually non-equally distributed in the city and not stable in time either, so it makes the final noise mapping integration a complex problem. The NoiseSPY project [58] designed a sound sensing system considering a mobile phone as a low-cost data logger to monitor environmental noise; citizens were allowed to visualize real-time noise levels in different places of the city. The NoiseTube project [59] provides a low-cost solution for the citizens to measure their personal exposure to noise in their everyday life by means of a mobile application that evaluates noise using the smartphone, allowing them to participate in the generation of a collective noise map using the geo-localized measurements. In [60], an application named NoiseMap is presented, which gathers loudness data and transfers them to an open urban sensing central platform. Afterwards, the data become public by means of a web-based service. In [61], the participative sensing contributes to updating a previous noise map of the city in order to dynamically refine the granularity of the noise patterns on different places. The frequency of updating depends on the level of participation of citizens in each road segment. Finally, [55] presents the OnoM@p system, which collects crowd noise data recorded by inexperienced volunteers by means of a smartphone application; the data are afterwards processed and filtered from outliers in the cloud, before being finally mapped and published on the web.

The Sounds Of The City project [62] is focused on the measurement of the noise level that surrounds a citizen. The citizen can measure the loudness of his/her environment using a simple smartphone application, which sends the data to a central server where all of the data are aggregated before computing and plotting the visual noise map. This idea was also exploited in a platform that modeled the noise situation of New York City considering three dimensions [63]: the region, the type of noise and a time stamp. From the collected data, a noise pollution indicator is inferred from the intelligent composition of noises measured by citizens.

Nowadays, this approach is gaining in importance since almost any smartphone can be used as a tool to sense citizens’ environment. On the one hand, authorities are interested in having real-time data measured in the city, especially in noise critical places, and on the other hand, citizens are interested in reporting noise excesses in their surroundings. One of the most recent trends in analyzing the acoustic data obtained from citizens is distinguishing between pleasant and unpleasant sounds, analyzing the relationship between soundscapes and emotions, as well as the relationship between soundscapes and people’s perceptions [64].

However, besides the complexity of the gathering and integration of such an amount of data, several voices [65] in the literature point out that the reliability of the derived noise maps depends on the quality of the smartphone’s microphone, plus the employed application, and the nature of the measure itself, which is conducted by any citizen at any place and at any time. Therefore, the accuracy of the measures cannot be guaranteed as if it were performed by experts using calibrated equipment. Other voices [66], supported by several studies comparing participative noise mapping with standard noise mapping techniques, maintain that the same accuracy can be achieved if the participative sensing is conducted properly.

### 2.4. Hybrid Urban Sensing

Nowadays, many projects are already designed to collect samples of acoustic signals in the city using the combination of more than one method. The most usual procedure is to take the basis to build the map from a static network in the city, with a small number of nodes of measurement. Then, they complement the measures with either mobile acoustic sensors or participative measurements made by volunteers.

In [67], they work in a Rotterdam urban neighborhood to detect noticeable sound events, because the perception of one’s acoustic environment in which they live is mainly driven by those. They used twelve intelligent sound measure devices, active for several months. At the same time, they collected citizens opinion about the sound in their home with a continuous survey. The final goal of the project was to report the relationship of between the detected events and the sound quality of the citizens. In [68], the same authors worked on a project in the 13th district of Paris using the data from twenty-four fixed sound measurement devices, while mobile sound measurements are performed in regular periods in the same area. The map is basically generated with the data of the fixed sensors, and it is changed with the mobile measurements accordingly.

## 3. Mobile Measure Platforms and Their Connectivity

There are two outstanding challenges of systems in charge of monitoring sensors that are located in remote areas or a net of sensors where nodes are moving in a dynamic topology: the first one it is to design the system in charge of collecting the information recorded by the sensors, reducing its price as much as possible. This issue would not be a problem when only a few sensors are needed, there are no power supply restrictions and the information processed by the sensors can be easily accessible by wired and inexpensive solutions, as happens normally in industrial applications. However, when the application requires a certain number of sensors and also a level of in situ real-time signal processing, this can be an important issue in the application design process.

The second challenge is to receive all of this information in a central system to post-process, store and take actions according to a predefined protocol of operation or to provide applications and functionalities to final users based on these data. As was mentioned before, industrial sensors are usually wired to Programmable Logic Controllers (PLC) or other complex systems, which deploy different communication interfaces. However, when the nodes are moving, the connectivity with the central system could be a great challenge if there are restrictions about radio frequency coverage and the cost of the communications (mainly when mobile communications are used to create these links with the nodes).

In the following subsections, these issues are analyzed taking into consideration the requirement of an embedded electronic system that is required to control the noise sensors used to create a low-cost network for monitoring in real time the acoustic environment in an urban scenario.

### 3.1. Hardware Platforms

Attending to the requirements mentioned previously, the electronic device that better satisfies them is a microcontroller-based embedded system. Nowadays, embedded systems can be found everywhere, from simply electronic applications to complex automation systems. Basically, an embedded system is a special purpose computer that has been designed for a specific function combining a software application and hardware controlled by a real-time operative system. These systems normally include soft-core processing units as microcontrollers, microprocessors, FPGAs, Digital Signal Processors (DSPs) and Application-Specific Integrated Circuits (ASICs) [69].

When talking about embedded systems, there is a huge variety of options in the market, but the selection can start by the core of the system, that is the microprocessor or the Central Processing Unit (CPU) of the embedded system. Nowadays, 32-bit microcontrollers are offered at a really competitive price and are included in almost all of the commercial embedded systems, as is shown in Table 1.

Table 1 only gathers a set of the most representative embedded systems used nowadays. All of the prices were obtained from common dealers, such as Farnell, Digikey and RS-online. Most of the embedded systems that are available on the market have a processor based on an ARM architecture, but the ARM^®^ Cortex^®^ series of cores considers a very wide range of options, offering designers a great deal of choice and the opportunity to use the best-fit core for their application [70]. There are three main categories of the Cortex Series:

- Cortex-A: basically used by embedded systems that need a high level of operational system computing capabilities as low-cost handsets to smartphones or tablets.
- Cortex-R: this series is the smallest ARM processor and is commonly used in automotive, networking and data storage applications.
- Cortex-M: being the most popular of the ARM family, this series is being used for all types of low-cost and low consumption applications, from real-time signal processing to industrial control.

Based on this analysis, the FRDM-KL25Z designed by NXP is considered as a good choice for the topic under discussion; that is, the processing of the acoustic environment in an urban scenario. This selection is done basically due to its low price, capability of processing analog signals in real time thanks to its 12-bit ADCs and its low power consumption (down to 47 μA/MHz), which makes it possible that the system could be powered by a portable battery pack. Furthermore, applications for this ultra-low-cost development platform can be designed using two very simple development environments:

- mbed: this is a platform that provides free libraries, hardware designs and online tools for rapid prototyping of 32-bit ARM-based microcontroller products. This framework includes a standards-based C/C++ SDK, a microcontroller HDK and supported development boards, an online compiler and online developer collaboration tools [71,72].
- MATLAB: MathWorks offers the Embedded Coder Support Package for the Freescale FRDM-KL25Z Board to run the Simulink^®^ model on an FRDM-KL25Z board. The support package includes a library of Simulink blocks for configuring and accessing Freescale FRDM-KL25Z peripherals and communication interfaces. Then, it is quite simple to build applications using the block-based interface of Simulink^®^, which generates also the code for the Freescale FRDM-KL25Z board and runs the generated code on the board [73].

Joined to the basic electronic elements of the embedded system, the application to which this paper is devoted requires an audio module to monitor the acoustic environment. Basically, in an embedded system, an audio module is based on a set of blocks, each one with a specific function [74]:

- Signal conditioning module, in charge of accommodating the analog signal captured by the microphone to the next block; that is, the Analog to Digital Converter (ADC). This conditioning stage adjusts the analog signal levels of voltage and current to the ADC input. Besides, this module also matches the output impedance of the microphone to the input impedance of the ADC.
- Analog to digital audio converter. Obviously, this block converts the analog signal captured by the microphone to a digital signal that can be later processed. To select the ADC, the main characteristics that should be analyzed are: the number of bits used in the conversion (as many bits are used, a bigger resolution will be obtained in the conversion, but a longer time of conversion), the speed of the conversion, distortion performance, sensitivity and errors in the conversion. The ADC can be implemented inside the System on Chip (SoC) on which the embedded system is based or also it can be placed externally, and it is controlled via an I2C or SPI bus.
- CODEC block: Although this block is optional, it can be used to reduce the number of bits needed to save the digitalized signal in the memory of the embedded system to its post-process. A huge variety of codecs can be used, but all of them are focused on compressing the audio stream with the maximum fidelity and quality.
- Memory: after the CODEC, data are dumped into a memory block from which the data can be retrieved for further processing by the application program.

In this application, the sensor needed to monitor the acoustic environment is a microphone, an acoustic-to-electric transducer that converts sound into an electrical signal. To select the best solution, one more advantage is found if the FRDM-KL25Z board is chosen, due to it featuring Arduino-compatible headers. Then, a great set of microphones can be used in the project. However, as it wanted to collect and log noise pollution, the sensor has to satisfy a set of characteristics, as:

- Directivity: as it is desired to monitor the noise generated in all directions, the microphone must be omnidirectional; that is, it must be able to capture noise from all directions, and then, it must be omnidirectional.
- Sensitivity: understood as the ratio of the analog output voltage or digital output value to the input noise pressure. This value, combined with the signal to noise ratio, is quite important to obtain a high quality monitoring system in order to record the sound with the maximum fidelity.
- Signal to noise ratio: it specifies the ratio of a reference signal to the noise level of the microphone output. Measured in decibels, it is the difference between the noise level and a standard 1-kHz, 94-dB SPL (Sound Pressure Level) reference signal. This specification is typically specified as an A-weighted value (dBA), which means that it includes a correction factor that corresponds to the human ear’s sensitivity to sound at different frequencies. Combined with the sensitivity, these factors will be important to be able to discriminate background noises during the monitoring of the acoustic environment.
- Operating frequency: this is the range of frequencies that can be collected by the microphone. As the application under design is to control the noise in an urban scenario and analyze its impact on humans, the frequency range should be from 20 to 20 kHz; that is, the dynamic range in with human ears work.

Taking into account these premises, the recommended solution is the elected microphone with auto again control based on the MAX9814 amplifier [75]. This microphone amplifier module is based on the CMA-4544PF-W omnidirectional capsule microphone, which provides a high sensitivity (−44 ± 2 dB), an operating frequency from 20 to 20 kHz and a signal to noise ratio of 60 dBA. Moreover, thanks to the MAX9814 amp, it performs automatic gain control, avoiding overwhelming and distortion of the amplifier when sound levels can change randomly, as in the scenario proposed in this paper.

### 3.2. Connectivity of the Platforms

Once the data are recorded and saved in the embedded system, they can be processed on-board or sent to a central system for being post-processed. Anyway, the embedded system must provide a wireless communication interface. Two alternatives are available to be deployed on the FRDM-KL25Z platform: a wireless and/or a 3G connection. With the adopted hardware, both solutions are possible at the same time, because the FRDM-KL25Z provides several universal asynchronous serial ports that can be used to communicate with both interfaces. The recommended hardware solutions are:

- WiFi ESP8266: this is a very low price and consumption WiFi module that implements a complete TCP/IP protocol stack. It provides a set of instructions and functionalities that make it very easy to control and start to work without any complicated configuration.
- Adafruit FONA 808: this is and all-in-one mobile communication interface plus a GPS module. Although it works with a 2 G SIM card, it is quite enough to allow remote control and data download, reducing the price of the whole system. It communicates with the controller through a serial port, making the deployment of applications easy and fast.

Thanks to these interfaces, the platform can be remotely accessed and controlled. The easiest and fastest way to implement this communication is deploying web services, which are transparent to the communication interface. The use of web servers is quite extended in the field of remote monitoring and controlling [76,77]. As in [78], the development of this application will be based on a Service-Oriented Architecture (SOA), in which most of the control is distributed in the server, which will access the remote sensing platform through SOAP messages to a web service developed for the control software solution.

The adoption of both interfaces increases the options of remote controlling. Basically, the WiFi interface will be used to upload stored data (acoustic samples) in the embedded system to the central system when a WiFi network is available. If the storage capacity of the embedded system is almost full, then the mobile interface would be used to send these data to the central system. The management of the communication interfaces and the selection of each one according to their availability will be done by the central processor of the embedded unit. Moreover, in all of the situations for which the central system wants to access the embedded system, the mobile communication interface assures this link.

**Figure 1:**
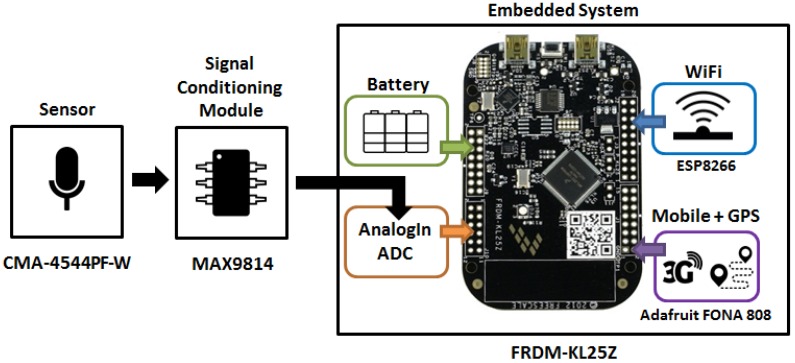
Suggested hardware platform. This is based on the FRDM-KL25Z embedded system. The microphone is the CMA-4544PF-W capsule followed by the MAX9814 amplifier. The platform also provides GPS, WiFi and mobile communications modules.

Therefore and according to the suggestions of this section, the complete hardware solution for monitoring the acoustic environment in the frame of a mobility scenario in a city is shown in Figure 1.

## 4. Mobile Bus Acoustic Measurement

The use of a public transport vehicle, such as a bus, to perform the measures presents evident advantages—power supply, mobility and connectivity—but also some specific drawbacks. In the literature, detailed in Section 2, there are several projects based on mobility to measure the noise in the city; on the one hand, deploying equipment temporally in vehicles (e.g., cars) to perform punctual measurements in specific locations of the city and, on the other hand, installing the sensors in non-motorized vehicles to get the vehicle to get the benefits of fast moving and maximum flexibility. In this paper, we propose a new scenario, in which a line bus carries the low-cost instruments, installed permanently for the realization of dynamic noise measurements in time. There are already previous experiences for the connectivity of urban buses [79,80], but mainly focused on fleet management, vehicle punctuality and the analysis of environmental parameters. Our proposal focuses on the possibility of evaluating dynamic noise maps in the city with a stable installation in their bus networks. This proposal faces new challenges, and for this purpose, in this section, we detail several challenges that a ubiquitous acoustic measurement system has to solve to allow a reliable dynamic acoustic mapping.

### 4.1. Signal Processing Challenges

There are several challenges in terms of signal processing when facing the mobile, bus acoustic measure of traffic noise levels. First, acoustic events different from the ones produced by traffic noise (hereafter, denoted as anomalous noise events) have to be detected and discarded to avoid biasing the $L_{eq}$ measurement of the traffic noise. Second, the noise generated by the mobile vehicle used to perform the measure has to be appropriately considered as traffic noise, but adjusting its contribution according to its closer distance to the point of measurement. The integration of input data in the server of the mobile sensor network is also an issue to be solved by means of noise mapping generation techniques; to tailor the most suitable noise map for every city, the chosen bus routes have to be previously studied so that they are representative enough and cover critical places in terms of noise. Finally, the classification of different type of vehicles is a real challenge in order to improve the information about traffic noise mapping, e.g., to distinguish between light and heavy traffic.

#### 4.1.1. Reliability of the $L_{eq}$ Measure

The goal of measuring the road traffic noise automatically faces inevitably the problem of dealing with non-traffic acoustic events (e.g., train, road work, bells, animals, human talking, etc.). An anomalous noise event detection algorithm has to be designed if we face the challenge of improving the robustness of the traffic noise mapping system. The challenge in this case is focused on identifying the most salient events to avoid their contribution to the $L_{eq}$ computation value.

Several works have been already conducted about this topic. In [81], a two-stage recognition schema is presented to detect abnormal events that take place in an usually noisy environment. In [82], the work is focused on detecting only police sirens recorded together with traffic noise. Both algorithms are based on Hidden Markov Models (HMM) working with Mel-Frequency Cepstral Coefficients (MFCCs). In [83], MFCCs are combined with Support Vector Machines (SVM) to identify hazardous situations, e.g., tire skidding and car crashes, in order to detect accidents. These methods follow a classic classification approach since the number of anomalous events is limited to a certain universe. In [84], acoustic summaries of several quiet and noisy areas are constructed using an automatic method, based on Self-Organizing Maps (SOM), including a validation stage of the local residents. The map is tuned to the sounds that are likely to be heard in a certain location by means of unsupervised training.

However, one of the key challenges when dealing with anomalous noise event detection is the definition of anomalous events themselves, i.e., any sound source different from traffic noise; hence, it cannot be limited to a finite number of categories a priori. In [85,86], an anomalous event detection algorithm is presented as a binary classifier, detecting whether the input acoustic data come from road traffic noise or if it is an anomalous event by considering Gammatone Cepstral Coefficients (GTCC) for audio signal parametrization [87]. Both K-Nearest Neighbor (KNN) and Fisher’s Linear Discriminant (FLD) performances are compared in [85] and in [86]. This algorithm can be performed in real time, just before the $L_{eq}$ computation, in order to avoid the integration of the anomalous events $L_{eq}$ in the noise map computation.

The main challenge of the anomalous event detection is to increase its accuracy when detecting the events, being capable of discarding those anomalous events to be detected that have not been yet observed during the training stage. As far as we know, this generalization has not been fully reached up to day, so it remains an open scientific challenge to be solved.

#### 4.1.2. Mobile Vehicle Noise Contribution

Another challenge associated with audio signal processing for ubiquitous noise measurements is the correction that has to be applied to the noise generated by the mobile vehicle transporting the acoustic sensor; in our case, a bus. Obviously, the bus itself contributes to traffic noise. However, this noise source is very close to the point of measure. Therefore, its has to be detected before filtering its contribution accordingly. Nevertheless, this problem presents a counterpart: the process can take advantage of having the audio reference of the noise source (mainly produced by the bus engine). In the following paragraphs, we describe some related approaches that can be considered to address the problem at hand.

In [88], a signal processing system dealing with identification and the estimation of the contribution of each source to an overall noise level is presented. The proposal is based on Fisher’s linear discriminant classifier and estimates the contribution based on a distance measure. Later, in [89], a system based on probabilistic latent component analysis is presented. This approach is based on a sound event dictionary where each element consists of a succession of spectral templates, controlled by class-wise HMMs.

In [90], the difficulties for appropriately measuring the performance of polyphonic sound event detection are stated before gathering several metrics specifically designed for this purpose. Sound event recognition complexity varies, and for the problem at hand of bus noise and traffic noise, the complexity is significant as, on the one hand, the sounds are continuously overlapped and, on the other hand, the type of signal to be detected is very similar. To this aim, a supervised model training for overlapping sound events based on an unsupervised source separation is presented in [91]. In [92], the authors detect sound events from real data using coupled matrix factorization of spectral representations and class annotations. Finally, in [93], the authors exploit deep learning methods to detect acoustic events by means of exploiting the spectro-temporal locality. For more references and detail, the reader is referred to [90].

In a nutshell, the most challenging issue for the problem at hand is integrating the bus and surrounding traffic noise levels to the noise map due to the similarity between the signal to be canceled and the signal to be processed to compute the $L_{eq}$ value. This increases the complexity of the separation system significantly, which may be addressed thanks to having the reference signal (i.e., considering as input the noise generated by the bus for the sound source separation).

#### 4.1.3. Classification of Road Traffic Vehicles

Finally, and despite it not being strictly necessary for dynamic noise mapping, there is the possibility of identifying the type and evolution of vehicles along time for a given area. This completes the information of the acoustic mapping, not only in terms of $L_{eq}$, but also in terms of type of traffic at each point of the city, e.g., by identifying the volume of heavy or light traffic [8].

In [94], a hierarchical classifier of urban sounds is proposed differentiating mechanical from non-mechanical sounds. The approach is based on MFCCs and HMM to perform the classification. In [95], the type of vehicle is also hierarchically classified besides extracting individual pass-by acoustic signatures of non-overlapping road traffic vehicles. In [96], the noise maps are refreshed using source separation from different types of vehicles (e.g., train, bus, car, etc.). In [97], an automatic sound recognition algorithm for the urban environment is detailed, reaching the best results for an SVM classifier. Finally, in [98], a complete review describing several methods to classify urban sounds is described. However, they are described under the umbrella of audio surveillance; the reviewed techniques are also applicable to the described challenge.

The challenge at this point is to classify a closed type of vehicle to characterize the type of traffic both at the high level (by means of noise maps) and low level (by means of sound source identification) across a particular urban area. These data may complete the information of the dynamic noise mapping services, allowing authorities to manage city traffic more precisely.

### 4.2. Challenges in Terms of Noise Mapping

In this section, two problems related to the design and tailoring of the noise maps are discussed. The first one deals with the problem of determining the most suitable locations that the mobile acoustic sensor should measure, i.e., the bus routes used to sense the city. The second one is related to the data gathering together with subsequent processing and integration to guarantee real-time noise mapping.

#### 4.2.1. Mobile Trajectories Design and Data Collection

In order to collect the suitable data to build the noise map from the acoustic information gathered in some city buses, previous field studies have to be conducted so as to choose which bus lines better fit the expected goal depending on their trajectory, the traffic volume, the authorities criteria, etc.

In [99], the authors collect a total amount of 4200 h of measurements, in 24-h block periods. This information is used to analyze the time patterns of the noise levels in certain places, but most relevantly, under several weather conditions.

In [100], the authors study if urban noise can be stratified by measuring traffic noise after dividing the streets of a particular city depending on their use on the type of street. The approach is tested and validated in five medium-sized Spanish towns. Later, the same authors studied to what extent this preliminary approach could be generalized to larger towns, showing a significant predictive capacity [101].

In [102], a statistically-based method to choose the optimal number and location of static monitoring sites to improve the noise mapping is presented, being focused to the development of noise maps with data obtained from a low-cost network. They present some preliminary results of the application of the method to the streets of Milan, improving the spatial sampling considering legislative road divisions, which are notonly based on geometry, but on noise emission.

The project will consider the study of the streets of the city to conclude the most suitable bus routes to conduct the traffic audio measurements. The system will collect traffic noise measurements, but as the hardware platform allows us to collect data from more sensors, we also intend to gather meteorological data, such as temperature and humidity. These allow one to widen the study of traffic noise variations taking into account different weather situations, in addition to the time noise level variations that a study of the whole day already provides. All of this gathered information allows us to perform comparative studies between the legislative classification of streets and acoustic measurement tests, pretending to obtain a classification of streets depending only on noise.

#### 4.2.2. Noise Mapping Real-Time Update

In the last years, several algorithms to address real-time noise mapping have been proposed in the literature, following three main approaches: (i) real-time calculation; (ii) map rescaling and sum and (iii) citizens’ contributions; a similar approach to the method described in Section 2.3.

In the real-time calculation approach, the measured values are introduced to the simulation software to evaluate a new value for the noise maps; the server has to reevaluate the entire noise map continuously, with a heavy computational load associated with that process. Gdansk University developed a real-time calculation approach system [103], and Ghent simplified the algorithms to make the evaluations faster [104].

The map scaling and sum approach is based on a similar infrastructure, but the noise mapping server evaluates the new noise map with the sum of the prescaled precalculated map according to the measured noise (i.e., it follows an incremental process). Hence, the computational load of the server is lower than the real-time approach. ACCON [105] uses an external GIS software technique to scale and sum the partial maps, and so does SADMAM (*Sistema de Actualización Dinámica del Mapa Acústico de Madrid*) [106]. In [106], the details of the software are given: the measurement data and GIS, the measurements integration to improve the source model, calibration of multiple sources in complex environments and also integrating GPS in the data. Finally, IDASC [107] scales and sums the partial maps using a function implemented inside the noise model software, avoiding the use of an external GIS, and publishes on the web new updated maps together with the noise data measured.

Finally, the citizens’ measurements using their smartphones also contribute to updating the information about noise levels in urban areas. In this case, the smartphone sends the data to the cloud with the GPS position. The aforementioned project NoiseTube [108] is an example of this type of platform, and Ear-Phone project [109] deals with the context-aware sensing, using classifiers to determine the sensing context of the phone; it allows to decide whether to sense or not automatically.

The proposed system, which is nowadays in a previous stage of development, will analyze the literature proposals, and the solution that fits best the dynamic of the measurements will be implemented to build the noise maps. However, the update time of the noise maps and how this is implemented are also open research issues. The noise maps should be refreshed only when there is a significant variation in the $L_{eq}$ value; the noise mapping update rhythm is calculated for each system depending on the time variation of the noise measurements 24 h a day in the places of interest. This study has to be conducted for the planned routes after the study detailed in Section 4.2.1, and the variation of the noise measurements values depending on the hour of the day has to be evaluated to conclude with the periodical update needed.

### 4.3. Challenges in Terms of Hardware Platform Selection

From the hardware platform point of view, the application under analysis presents the following challenges:

- Low price: the main goal of the application being the collection of data about the noise in the city to generate a dynamic noise map, the more sensors can be placed, the better. Then, the use of a inexpensive hardware platforms is recommended, which can be easily deployed, and this should include all of the elements needed to achieve the objectives of the applications.
- Non-intrusive: that is, as the platform will be deployed on a public means of transport, such as urban buses, it is recommended not to require any special restrictions regarding its installation. Then, it must be auto-powered, small in size and easily integrated on the buses. Moreover, during the performance of the system, it has to have no interference with the electronic and communication systems of the vehicle.
- Low energy consumption: as the hardware platform cannot be connected to the energy system of the vehicle, it must have a power system itself. Then, the complete system should be low consumption and implemented with any software functionality able to control the waste of energy during the operation, for example disabling some subsystems if they are not needed.
- Communication interfaces: one of the main tasks of the platform is to send the recorded data to a central system. Then, different communication interfaces can be deployed on board, according to the characteristics of the environment. That is, if a WiFi network is available, then a free link can be established with the server, but in the case of being out of the WiFi coverage, a mobile communication link should be available to allow the data upload and also the remote control of the platform in case a remote reconfiguration or maintenance is necessary. At the same time, the platform must perform a geolocation interface in order to associate noise-data to location references.
- Communications management sub-system: as the platform needs different interfaces, a management software is needed to control the communications to the server. This functionality will be in charge of determining which interface can be used in each situation in order to save energy and to save money. For example, when free WiFi is available, this functionality will upload all of the saved data to the server automatically.
- Storage capacity: as the platform must save data about the noise in different locations, it must be able to save as much information as possible in order to keep it in memory until being in a free WiFi coverage area to send it to the server or just before overloading of the memory, through a mobile link.
- Real-time data processing capability: the processor provided on the embedded system must be able to process the noise captured by the microphone in real time to obtain the noise frequency and level. Then, this information is saved on the memory joined to the location provided by the GPS. Later, these data are sent to the central server. During this signal processing stage, the processor also must be able to discriminate the noise of the environment from the noise generated by the vehicle in which the hardware will be deployed. Moreover, the resolution of the processing must be enough to allow the classification and characterization of the traffic vehicles.

In summary, the hardware platform chosen for creating the dynamic noise map must be easily installed in a public means of transport like the buses of a city. It should be as inexpensive as possible to allow multiple instances of the platform in different vehicles, obtaining information about the noise in all of the areas of the city. It must deploy different communication interfaces with the aim of allowing the upload of recorded data to the central server. At the same time, the platform must perform real-time data processing to obtain as much information as possible about the noise and to detect and filter the vehicle audio contribution, in which the hardware will be deployed.

## 5. Conclusions

This work describes the design of a mobile approach for ubiquitous urban noise sensing using public buses. It widens the input data coverage with respect to static approaches, since it allows sensing the city by means of several bus lines carrying acoustic low-cost sensing platforms. Hence, due to the fact that the vehicle moves across the city, the noise map is fed with ubiquitous measurements. Moreover, when compared to other mobile acoustic sensing systems, it takes advantage, on the one hand, of the possibility of changing the measurement route by just selecting another bus line to carry the sensor and, on the other hand, of using a vehicle where the hardware can be installed and powered easily. In regards to participative sensing, a closed route repeated several times a day should guarantee enough data to build a reliable noise map, and it solves the heterogeneity and the accuracy problems when integrating data from citizens’ measurements (i.e., different types of smartphones, under- or over-sampled locations, etc.).

Nevertheless, before implementing the proposed approach, several challenges should be addressed; signal processing challenges, to work with the bus engine noise and its contribution to the noise map, which nowadays is an open scientific topic; noise mapping challenges, to study which are the best bus routes for the measurements, and the proper building of the noise maps in the cloud from ubiquitous acoustic data. Finally, hardware challenges face the design of the sensor in a low-cost and low-power consumption platform, but with enough computational capacity to implement the necessary signal processing algorithms to perform the measures and afterwards send them to the cloud. These are key factors in all systems that will be deployed under the paradigm of smart cities, since these systems should be autonomous and transparent, by requiring easy and occasional maintenance and minimizing the interference with the other elements of the city, as in the scenario proposed in this work, with the public transport system. The long-term vision of the implementation of the proposed system is that the entire city, or at least its major roads, will be mapped through the proposed mobile noise sensing system.

In the near future, we plan to address the aforementioned challenges before implementing and evaluating the proposal in a real-life scenario.

## Figures and Tables

**Table 1:** Embedded systems’ comparison.

Embedded System	Processor Core	Price
The chipKIT™ MX3	Microchip^®^ PIC32MX320F128H Microcontroller (80-MHz 32-bit MIPS 128 KB Flash, 16 KB SRAM)	44.99$
STM32VLDiscovery	ARM^®^ Cortex-M3 (24-MHz 32-bit 128 KB Flash memory, 8 KB RAM)	9.90$
FRDM-KL25Z	ARM^®^ Cortex^®^-M0+ (48-MHz 32-bit MIPS 128 KB Flash 16 KB SRAM)	13.25$
BeagleBone Black	Sitara™ ARM^®^ Cortex-A8 (2x PRU 32-bit microcontrollers, 512 MB DDR3 RAM)	51.15$
Raspberry Pi 3 Model B	1.2-GHz Quad-Core ARM Cortex-A53	37.00$
CYPRESS PSoC^®^ 4 CY8C4245AXI	32-bit ARM^®^ Cortex™-M0 48-MHz CPU	24.31$

## Abbreviations

The following abbreviations are used in this manuscript:

ADC	Analog to Digital Converter
ANED	Anomalous Noise Event Detection
ARM	Advanced RISC Machine
ASIC	Application-Specific Integrated Circuits
CPU	Central Processing Unit
EC	European Commission
END	Environmental Noise Directive
EU	European Union
DSP	Digital Signal Processor
FLD	Fisher’s Linear Discriminant
FPGA	Field Programmable Gate Array
GPS	Global Positioning System
GTCC	Gammatone Cepstrum Coefficients
HMM	Hidden Markov Models
ICT	Information and Communication Technology
KNN	K-Nearest Neighbor
LMS	Least Mean Squares Filter
MFCC	Mel-Frequency Cepstrum Coefficients
MSU	Mobile Sensing Unit
PLC	Programmable Logic Device
RAM	Random Access Memory
RLS	Recursive Least Squares Filter
SOA	Service-Oriented Architecture
SoC	System on Chip
SOM	Self-Organizing Maps
SPL	reference signal
SVM	Support Vector Machine
WASN	Wireless Acoustic Sensor Network
